# Supplementary material for: Detection of SARS-CoV-2 in subcutaneous fat but not visceral fat, and the disruption of fat lymphocyte homeostasis in both fat tissues in the macaque
Source: Commun Biol. 2022 Jun 3;5:542. doi: 10.1038/s42003-022-03503-9 (PMC9166782; doi:10.1038/s42003-022-03503-9)

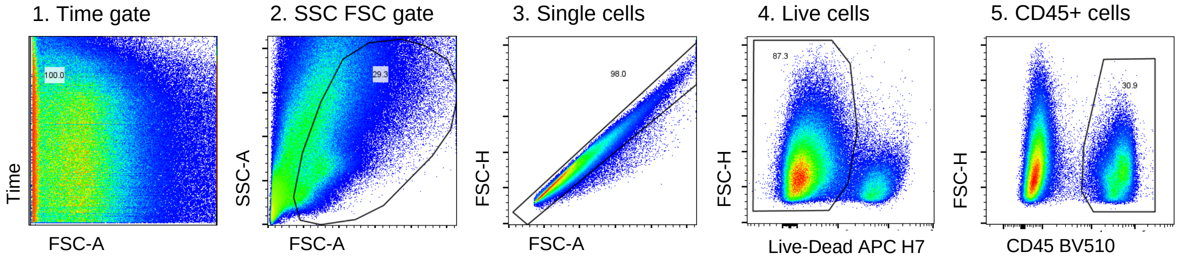

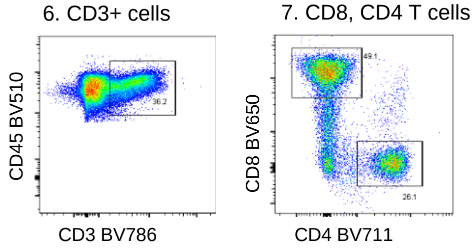

Supplemental Figure 1. Gating strategy.

Gating strategy for stromal vascular cells collected after dissociation of VAT sampled from a cynomolgus macaque infected with SARS-CoV-2. The quality of the cell suspension was checked using the time vs. FSC gating. Next, cells are selected in a broad FSC/SSC gate, from which doublets and dead cells are subsequently excluded. Staining with an anti-CD45 antibody selects hematopoietic cells, which are then stained for CD3, CD4, and CD8.

Supplemental Figure 2. CD69 expression among CD4 and CD8 T cells in blood.

Proportions of CD69⁺ CD4 T cells and CD8 T cells as determined by flow cytometry. The graphs show data from uninfected NHPs (black open circles: n=4 for SCAT, n=5 for VAT). Each symbol represents a distinct animal. The median [IQR] proportion of CD69⁺ cells in blood among CD4+ cells and CD8+ cells was 5.9% [4.9; 7.6] and 0.8% [0.2; 1.4] respectively in the infected group and 2.2% [0.9; 3.6] and 5.7% [2.8; 10.7] respectively in the control group. Statistically significant differences (as determined in a nonparametric, unpaired Mann–Whitney test) are indicated as follows: *$p < 0.05$).

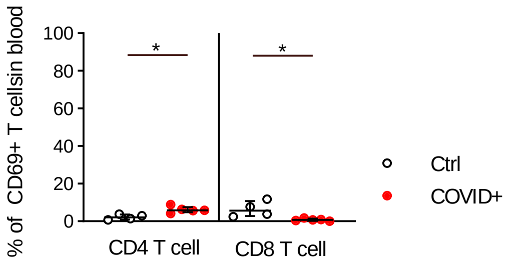

Supplemental Figure 3. Determination of T cell numbers in the blood during the course of the infection

Numbers of lymphocytes cells as determined using a HMX A/L analyzer (Beckman Coulter). **Dynamic of lymphocyte number during SARS-CoV-2 infection.** Each symbol represents a distinct animal. The values were compared in a Friedman test with a Dunn's multiple comparison post-test. Statistically significant differences are indicated as follows: *$p < 0.05$, *$p < 0.01$

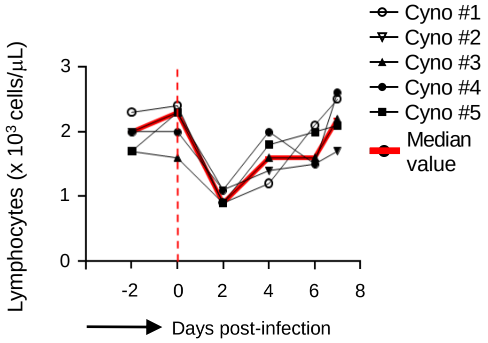

Supplement: Supplementary file 2 — Supplemental information [file 42003_2022_3503_MOESM2_ESM.pdf]
