# Supplementary material for: Detection of SARS-CoV-2 in subcutaneous fat but not visceral fat, and the disruption of fat lymphocyte homeostasis in both fat tissues in the macaque
Source: Commun Biol. 2022 Jun 3;5:542. doi: 10.1038/s42003-022-03503-9 (PMC9166782; doi:10.1038/s42003-022-03503-9)
Supplement: Supplementary file 3 — Description of Additional Supplementary Files [file 42003_2022_3503_MOESM3_ESM.pdf]

Description of Additional Supplementary Files

File name: Supplementary Data 1
Description: Raw data.
