# Supplementary material for: Detection of SARS-CoV-2 in subcutaneous fat but not visceral fat, and the disruption of fat lymphocyte homeostasis in both fat tissues in the macaque
Source: Commun Biol. 2022 Jun 3;5:542. doi: 10.1038/s42003-022-03503-9 (PMC9166782; doi:10.1038/s42003-022-03503-9)
Supplement: Supplementary file 5 — Reporting Summary [file 42003_2022_3503_MOESM5_ESM.pdf]

nature portfolio

Corresponding author(s): Christine BOURGEOIS

Last updated by author(s): April, 3,2022

Reporting Summary

Nature Portfolio wishes to improve the reproducibility of the work that we publish. This form provides structure for consistency and transparency in reporting. For further information on Nature Portfolio policies, see our Editorial Policies and the Editorial Policy Checklist.

Statistics

For all statistical analyses, confirm that the following items are present in the figure legend, table legend, main text, or Methods section.

n/a	Confirmed	
☐	☒	The exact sample size (n) for each experimental group/condition, given as a discrete number and unit of measurement
☐	☒	A statement on whether measurements were taken from distinct samples or whether the same sample was measured repeatedly
☐	☒	The statistical test(s) used AND whether they are one- or two-sided<br>*Only common tests should be described solely by name; describe more complex techniques in the Methods section.*
☒	☐	A description of all covariates tested
☐	☒	A description of any assumptions or corrections, such as tests of normality and adjustment for multiple comparisons
☐	☒	A full description of the statistical parameters including central tendency (e.g. means) or other basic estimates (e.g. regression coefficient) AND variation (e.g. standard deviation) or associated estimates of uncertainty (e.g. confidence intervals)
☒	☐	For null hypothesis testing, the test statistic (e.g. F, t, r) with confidence intervals, effect sizes, degrees of freedom and P value noted<br>*Give P values as exact values whenever suitable.*
☒	☐	For Bayesian analysis, information on the choice of priors and Markov chain Monte Carlo settings
☒	☐	For hierarchical and complex designs, identification of the appropriate level for tests and full reporting of outcomes
☒	☐	Estimates of effect sizes (e.g. Cohen's d, Pearson's r), indicating how they were calculated

Our web collection on statistics for biologists contains articles on many of the points above.

Software and code

Policy information about availability of computer code

Data collection	LSR Fortessa flow cytometer (BD Biosciences), CFX 96 Thermocycler (BioRad) Bioplex 200 analyzer (BioRad)
Data analysis	FlowJo software (version 10.6.2, FlowJo LLC, Ashland, OR, USA), GraphPad Prism software (version 8, GraphPad Software Inc., San Diego, CA, USA), BioRAD CFX Maestro (BioRad)

For manuscripts utilizing custom algorithms or software that are central to the research but not yet described in published literature, software must be made available to editors and reviewers. We strongly encourage code deposition in a community repository (e.g. GitHub). See the Nature Portfolio guidelines for submitting code & software for further information.

Data

Policy information about availability of data

All manuscripts must include a data availability statement. This statement should provide the following information, where applicable:

- Accession codes, unique identifiers, or web links for publicly available datasets
- A description of any restrictions on data availability
- For clinical datasets or third party data, please ensure that the statement adheres to our policy

All data generated or analysed during this study are included in this published article (and its supplementary information files).

Field-specific reporting

Please select the one below that is the best fit for your research. If you are not sure, read the appropriate sections before making your selection.

☒ Life sciences ☐ Behavioural & social sciences ☐ Ecological, evolutionary & environmental sciences

For a reference copy of the document with all sections, see nature.com/documents/nr-reporting-summary-flat.pdf

Life sciences study design

All studies must disclose on these points even when the disclosure is negative.

Sample size	The size of the study was defined by both ethical and statistical factors. Based on prior studies on NHP in SARS-CoV-2, a number of 5 animals is considered as ethically acceptable and statistically sufficient.
Data exclusions	No exclusion were applied on data, although some samples were too small to complete the whole analyses. If so, it is specified in the manuscript
Replication	For RT-PCR, the assays were carried out in duplicates. For cytometry, the antibody combination has been validated beforehand on other animals. Given the small amount of material available and the work on fresh sampling, we were not able to perform duplicates.
Randomization	Infected animals were randomized according to gender, age, weight, and infectious status.
Blinding	Blinding is not relevant in this study, animals were comparable among each group.

Reporting for specific materials, systems and methods

We require information from authors about some types of materials, experimental systems and methods used in many studies. Here, indicate whether each material, system or method listed is relevant to your study. If you are not sure if a list item applies to your research, read the appropriate section before selecting a response.

Materials & experimental systems

n/a	Involved in the study	
☐	☒	Antibodies
☒	☐	Eukaryotic cell lines
☒	☐	Palaeontology and archaeology
☐	☒	Animals and other organisms
☐	☒	Human research participants
☐	☒	Clinical data
☒	☐	Dual use research of concern

Methods

n/a	Involved in the study	
☒	☐	ChIP-seq
☐	☒	Flow cytometry
☒	☐	MRI-based neuroimaging

Antibodies

Antibodies used	CD45 (BV510, D058-1283), HLA-DR (BV605, G46-6), CD8 (BV650, RPA-T8), CD4 (BV711, L200), CD3 (BV786, SP34-2), CD69 (FN50, PercPCy5.5), from BD Biosciences; and PD-1 (BV421, EH12-2H7) from Biolegend (San Diego, CA, USA).
Validation	All antibodies were validated by manufacturer's website for flow cytometry application. Reactivity was validated for cynomolgus species by manufacturer's website for : CD45, CD3, HLA-DR, CD8, CD4, CD69. Reactivity for cynomolgus was validated by "Non Human Primate reagent" website for PD-1 and Ki67.

Animals and other organisms

Policy information about studies involving animals; ARRIVE guidelines recommended for reporting animal research

Laboratory animals	Animals were cynomolgus macaques (Macaca fascicularis). The infected group comprised five females. The median (interquartile range (IQR) age was 7.6 years [7.6; 7.7]. The uninfected (control) group also comprised five females (median age: 5.4 years [5.4; 7.0].
Wild animals	Adult cynomolgus macaques (Macaca fascicularis) were imported from AAALAC-certified breeding centers in Mauritius.
Field-collected samples	N/A
Ethics oversight	After a 1-year period of quarantine in Spain, animals were housed at the IDMIT facility (CEA, Fontenay-aux-Roses, France) in BSL-2 and BSL-3 containment conditions, when necessary). At the end of the study, infected animals were humanely euthanized with intravenous pentobarbital sodium during anesthesia with tiletamine and zolazepam, and an autopsy was performed. The

experiments were approved by an institutional review board (Comité d'Ethique en Expérimentation Animale du Commissariat à l'Energie Atomique et aux Energies Alternatives, Fontenay-aux-Roses, France; reference: CEtEA #44)).

Note that full information on the approval of the study protocol must also be provided in the manuscript.

Human research participants

Policy information about studies involving human research participants

Population characteristics	No covariates as data were obtained from a single group of SARS-CoV-2 non-infected patients.
Recruitment	Adipose tissue collection was performed either in the course of bariatric surgery or during surgery unrelated to weight disorders (e.g. cholecystectomy).
Ethics oversight	The study protocol was approved by the regional investigational review board (Comité de Protection des Personnes Ile-de-France VII, PP12-021, Paris, France).

Note that full information on the approval of the study protocol must also be provided in the manuscript.

Clinical data

Policy information about clinical studies

All manuscripts should comply with the ICMJE guidelines for publication of clinical research and a completed CONSORT checklist must be included with all submissions.

Clinical trial registration	N/A
Study protocol	N/A
Data collection	N/A
Outcomes	N/A

Flow Cytometry

Plots

Confirm that:

☒ The axis labels state the marker and fluorochrome used (e.g. CD4-FITC).

☒ The axis scales are clearly visible. Include numbers along axes only for bottom left plot of group (a 'group' is an analysis of identical markers).

☒ All plots are contour plots with outliers or pseudocolor plots.

☒ A numerical value for number of cells or percentage (with statistics) is provided.

Methodology

Sample preparation	Stromal vascular fraction was isolated from fresh AT samples. When necessary, the AT was devascularized before dissociation. AT was rinsed in Dulbecco's Modified Eagle's Medium (DMEM) (Lonza, Basel, Switzerland) with 5% fetal bovine serum (FBS), weighed, and cut into pieces of 2 to 3 mm (to optimize enzymatic digestion). The pieces were digested in a solution of type VIII collagenase from Clostridium histolyticum (0.33 mg/mL in DMEM with 5% FBS; Sigma-Aldrich, St. Louis, MO, USA). Enzymatic digestion was performed for 30 min at 37°C with constant stirring. This was followed by mechanical dissociation by repeated suction-expulsion of the suspension with a 10 mL syringe. The AT suspension was filtered through a cell strainer (pore size: 100 µm; Corning, New York, NY, USA) and centrifuged at 330 g for 8 min at room temperature. The upper phase of the supernatant was discarded, and the lower phase (comprising the SVF cells) was centrifuged at 660 g for 8 min at room temperature. The supernatant was discarded, and the pellet containing the SVF was filtered through a cell strainer (pore size: 100 µm) and resuspended in DMEM with 5% FBS. Trypan blue-treated cell suspensions were then counted under a microscope using a Malassez cell (C-chip, NanoEntek, Seoul, Korea). The SVF was either directly stained for flow cytometry analysis or cryopreserved in 90% FBS/10% DMSO.
Instrument	LSR Fortessa flow cytometer (BD Biosciences)
Software	FASC DIVA software for acquisition and FlowJo software (version 10.6.2, FlowJo LLC, Ashland, OR, USA).
Cell population abundance	The population of interest was selected using a gating strategy allowing exclusion of cellular debris (FSC SSC gate), doublets (FSC-A/FSC-H), dead cells (live dead staining). Immune cell susbets were selected with specific antibody staining (CD45, CD3, CD4 and CD8). Proportions of cells are indicated only if more than 300 cells were available in the parent subset.

Gating strategy

The quality of the cell suspension was checked using the time vs. FSC gating. Next, cells are selected in a broad FSC/SSC gate, from which doublets and dead cells are subsequently excluded. Staining with an anti-CD45 antibody selects hematopoietic cells, which are then stained for CD3, CD4, and CD8.

☒ Tick this box to confirm that a figure exemplifying the gating strategy is provided in the Supplementary Information.
